# 326. Optimizing DNA Extraction from Pediatric Stool for Diagnosing Tuberculosis and Use in Next Generation Sequencing

**DOI:** 10.1093/ofid/ofac492.404

**Published:** 2022-12-15

**Authors:** Tara Ness, Lennard A Meiwes, Alexander Kay, Rojelio Mejia, Christoph Lange, Anna Mandalakas, Andrew DiNardo

**Affiliations:** Baylor College of Medicine/Texas Childrens Hospital, Houston, Texas; Research Center Borstel, Lübeck, Schleswig-Holstein, Germany; Baylor Center of Excellence, Mbabane, Hhohho, Swaziland; Baylor College of Medicine/Texas Childrens Hospital, Houston, Texas; Research Center Borstel, Clinical Infectious Diseases, Borstel, Hamburg, Germany; Baylor College of Medicine/Texas Childrens Hospital, Houston, Texas; Baylor College of Medicine, Houston, Texas

## Abstract

**Background:**

Next generation sequencing (NGS) is quickly coming to the forefront of diagnostic tools to provide efficient, highly informative information from patient samples. Recently, it was established that patients with pulmonary Tuberculosis (TB) have *Mycobacterium tuberculosis* DNA present in their stool samples, which can augment current diagnostic gaps. Optimizing extraction of DNA from stool for analysis via sequencing technologies is a paramount initial step to ensure accuracy of downstream sequencing applications.

**Methods:**

Attenuated strains of *Mycobacterium bovis* derived from BCG were used as a model for Mtb. Human stool samples were spiked with varying known concentrations of BCG and extracted with four different DNA extraction kits (Fast DNA Spik Kit for Soil, DNeasy Blood and Tissue Kit, MagAttract HMW DNA Kit, and PowerFecal Pro DNA Kit). Each sample was subjected to quantitative polymerase chain reaction using designed primers and probes specific for identifying Mtb infection from stool. The samples underwent further analysis to assess overall DNA yield (Qubit fluorometer), DNA fragment length (Agilent tape measure), and DNA purity (Nanodrop spectrophotometer).

**Results:**

Overall, the Fast DNA Spin Kit for Soil extraction kit showed the most optimal results. DNA isolated via this method showed the lowest cycle thresholds of Mtb amplification, indicating the most preserved amount of BCG specific DNA. In addition, this method showed the highest overall DNA yield and highest proportion of long DNA fragment lengths. Fluorometric analysis showed significant contamination in the 230 nm wavelength range, which was amended with an additional AMPure bead cleanup step.

Quantiative PCR of Spiked BCG

**Figure:**
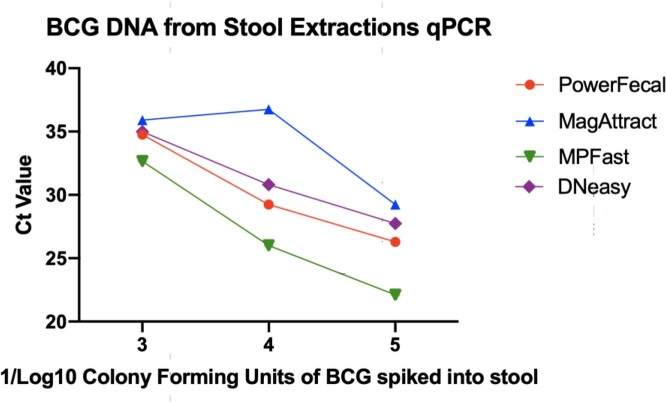

Quantitative PCR Cycle Threshold Values by Different DNA Extraction Kits

**Conclusion:**

The MPFast Soil Extraction kit, when compared to three other DNA extraction kits, performed the best on stool samples for isolating BCG DNA. Overall DNA yield, DNA length, and amount of specific BCG DNA were best optimized with this method and provided the best samples for sequencing analysis. This critical step is the first of many to realize the promise of stool-based NGS.

DNA Quantity

**Figure:**
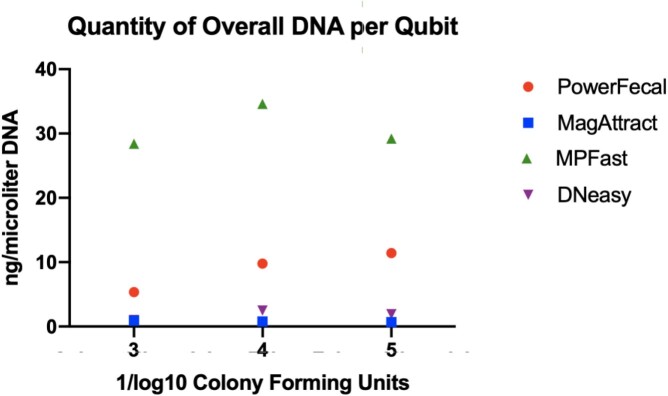

Quantity of Total DNA by Different DNA Extraction Kits

Spectrophotometric Analysis

**Figure:**
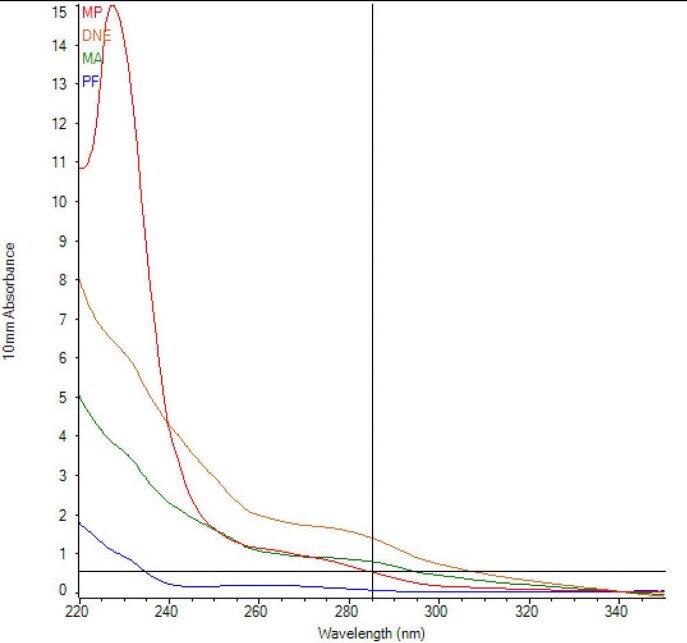

Analysis of Absorption of Extracted DNA by Different DNA Extraction Kits

Spectrophotometric Results

Spectrophotometry Results of DNA Samples Extracted by Different DNA Extraction Kits

**Disclosures:**

**Lennard A. Meiwes, n/a**, German Center for Infection Research (DZIF): Grant/Research Support.

